# Radiotherapy in the Management of Pediatric and Adult Osteosarcomas: A Multi-Institutional Cohort Analysis

**DOI:** 10.3390/cells10020366

**Published:** 2021-02-10

**Authors:** Mateusz Jacek Spałek, Jan Poleszczuk, Anna Małgorzata Czarnecka, Monika Dudzisz-Śledź, Aleksandra Napieralska, Jacek Matysiakiewicz, Marzanna Chojnacka, Anna Raciborska, Aleksandra Sztuder, Adam Maciejczyk, Agata Szulc, Tomasz Skóra, Bożena Cybulska-Stopa, Tomasz Winiecki, Joanna Kaźmierska, Bartłomiej Tomasik, Jacek Fijuth, Piotr Rutkowski

**Affiliations:** 1 Department of Soft Tissue/Bone Sarcoma and Melanoma, Maria Sklodowska-Curie National Research Institute of Oncology, 02-781 Warsaw, Poland; am.czarnecka@pib-nio.pl (A.M.C.); monika.dudzisz-sledz@pib-nio.pl (M.D.-Ś.); piotr.rutkowski@pib-nio.pl (P.R.); 2 Department for Computational Oncology, Maria Sklodowska-Curie National Research Institute of Oncology, 02-034 Warsaw, Poland; jan.poleszczuk@pib-nio.pl; 3 Nalecz Institute of Biocybernetics and Biomedical Engineering, Polish Academy of Sciences, 02-109 Warsaw, Poland; 4 Mossakowski Medical Research Centre, Department of Experimental Pharmacology, Polish Academy of Sciences, 02-106 Warsaw, Poland; 5 Department of Radiotherapy, Maria Sklodowska-Curie National Research Institute of Oncology, Gliwice Branch, 44-102 Gliwice, Poland; olanapieralska@gmail.com; 6 Trauma and Orthopedic Surgery Department, IXth Ward of the District Hospital of Orthopedics and Trauma Surgery in Piekary Slaskie, 41-940 Piekary Slaskie, Poland; doc76@poczta.fm; 7 Department of Oncology and Radiotherapy, Maria Sklodowska-Curie National Research Institute of Oncology, 02-034 Warsaw, Poland; marzanna.chojnacka@pib-nio.pl; 8 Department of Oncology and Surgical Oncology for Children and Youth, Institute of Mother and Child, 01-211 Warsaw, Poland; anna.raciborska@hoga.pl; 9 Department of Radiotherapy, Lower Silesian Oncology Centre, 53-413 Wroclaw, Poland; a.sztuder@gmail.com (A.S.); adam.maciejczyk@dco.com.pl (A.M.); agaszul@gmail.com (A.S.); 10 Department of Oncology, Faculty of Medicine, Wroclaw Medical University, 50-367 Wrocław, Poland; 11 Department of Radiotherapy, Maria Sklodowska-Curie National Research Institute of Oncology, Kraków Branch, 31-115 Kraków, Poland; tbskora@gmail.com; 12 Department of Clinical Oncology, Maria Sklodowska-Curie National Research Institute of Oncology, 31-115 Kraków, Poland; bcybulskastopa@vp.pl; 13 Radiotherapy Department II, Greater Poland Cancer Centre, 61-866 Poznan, Poland; to.winiecki@gmail.com (T.W.); joanna.kazmierska@wco.pl (J.K.); 14 Electroradiology Department, University of Medical Sciences, 61-701 Poznan, Poland; 15 Department of Radiotherapy, Medical University of Lodz, 92-215 Lodz, Poland; bartektomasik@gmail.com (B.T.); jacek.fijuth@umed.lodz.pl (J.F.); 16 Department of Biostatistics and Translational Medicine, Medical University of Lodz, 95-513 Lodz, Poland; 17 Postgraduate School of Molecular Medicine, Medical University of Warsaw, 02-091 Warsaw, Poland

**Keywords:** sarcoma, radiotherapy, osteosarcoma, radiotherapy, intensity-modulated, radiotherapy, image-guided, bone cancer

## Abstract

Background: Due to the rarity of osteosarcoma and limited indications for radiotherapy (RT), data on RT for this tumor are scarce. This study aimed to investigate the utilization of RT for osteosarcomas in the recent 20 years and to identify factors related to patients’ response to radiation. Methods: We performed a retrospective analysis of patients irradiated for osteosarcoma treatment. We planned to assess differences in the utilization of RT between the periods of 2000–2010 and 2011–2020, identify the risk factors associated with local progression (LP), determine whether RT-related parameters are associated with LP, and calculate patients’ survival. Results: A total of 126 patients with osteosarcoma who received 181 RT treatments were identified. We found a difference in RT techniques between RT performed in the years 2000–2010 and that performed in the years 2011–2020. LP was observed after 37 (20.4%) RT treatments. Intent of RT, distant metastases, and concomitant systemic treatment affected the risk of LP. Five-year overall survival was 33% (95% confidence interval (26%–43%)). Conclusions: RT for osteosarcoma treatment has evolved from simple two-dimensional palliative irradiation into more conformal RT applied for new indications including oligometastatic and oligoprogressive disease. RT may be a valuable treatment modality for selected patients with osteosarcoma.

## 1. Introduction

Osteosarcoma, a rare primary bone tumor, mostly affects children and young adults. It requires a multidisciplinary management. The standard treatment of localized disease consists of surgery with perioperative multiagent chemotherapy [1,2]. Radiotherapy (RT) is not used routinely for osteosarcoma due to the assumed radioresistance of osteosarcoma cells and advances in orthopedics surgery that have allowed extensive bone resections with wide margins followed by implantation of prostheses [3,4]. RT may be used in adjuvant treatment after surgery with positive margins and as a definitive treatment in the case of unresectable localized disease [5]. Furthermore, as with other cancers, irradiation could help palliate symptoms related to primary tumor or distant metastases. RT can be also an alternative to surgery in the presence of single metastases; however, this approach is not based on results of prospective studies [6]. Due to the predictable osteosarcoma radioresistance, RT treatment for this tumor requires a higher dose compared with treatment for other malignancies. The administered RT total dose is tailored to the achieved surgical margins and the proximity of organs at risk.

The delivery of high dose to target volumes may be greatly limited in osteosarcoma due to the usually large size of primary tumors and their frequent localization in anatomically challenging regions such as the pelvis or the paraspinal area. Introduction of modern dynamic RT techniques, namely, intensity-modulated RT (IMRT) and volumetric-modulated arc therapy (VMAT), have increased the dose conformity within the target volume with the simultaneous protection of the organs at risk. Unique properties of charged particles, such as protons and carbon ions, provide even a more favorable dose distribution, allowing the administration of high-dose RT to anatomically challenging regions. Moreover, distinct radiobiological features of the carbon ions may overcome the resistance of osteosarcoma cells [7,8].

Contemporary RT might be an underestimated treatment modality for patients with locally advanced or metastatic osteosarcoma, given that, currently, there are no large datasets on the utilization of modern RT for osteosarcoma patients, especially those with oligometastatic and oligoprogressive disease. The available literature data describe patients treated decades ago with older RT techniques [9,10].

The objectives of this study were to investigate the utilization of RT in the management of pediatric and adult osteosarcomas in the last 20 years and to identify factors related to relatively better responses to RT. We report the results of a multi-institutional cohort analysis.

## 2. Materials and Methods

### 2.1. Data Extraction

A retrospective multicenter analysis of pediatric and adult patients treated in the seven Polish national cancer centers between 2000 and 2020 was performed. We included all consecutive patients who underwent RT for primary, recurrent, or metastatic osteosarcoma. Medical records were screened individually in each participating center. All extracted data were centrally verified by the two authors from the sarcoma tertiary center (MJS, AMC) and recorded in the OpenClinica internal server.

### 2.2. Analyzed Parameters

The following parameters were included in the analysis: date of primary diagnosis, primary-tumor characteristics and site, the given curative treatment, indication for RT, performance status during RT, irradiated volume, concomitant systemic therapy, RT technique, total dose, equivalent dose in 2 Gy fractions (EQD2), dose per fraction, early and late RT toxicity, local response (best result and the incidence of in-field local progression), date and reason of death (if applicable). The missing dates of deaths were obtained from the Polish National Cancer Registry.

### 2.3. Data Interpretation

For the purposes of this analysis, we divided patients into two subgroups, i.e., those treated using RT with palliative intent and the others treated with non-palliative intent. Oligometastatic disease and oligoprogression were defined in compliance with the consensus recommendation provided by the European Society for Radiotherapy and Oncology and the European Organisation for Research and Treatment of Cancer [11]. The Eastern Cooperative Oncology Group (ECOG) scale was used to classify patients’ performance status during RT. We assumed the alpha/beta ratio of osteosarcomas as 4 to calculate EQD2, based on literature data suggesting its values for sarcomas between 0.4 and 5 Gy [12,13,14]. RT-related toxicity was reassessed using Common Terminology Criteria for Adverse Events 5.0 (CTCAE). Best local response and the incidence of local progression were assessed with Response Evaluation Criteria in Solid Tumors 1.1 (RECIST 1.1). In the case of imaging data unavailability, the local response was considered a stable disease, unless clinically apparent disease progression was observed.

### 2.4. Endpoints

To investigate our objectives, we planned to: (1) assess differences in the utilization of RT for patients treated with palliative and non-palliative intent between the periods of 2000–2010 and 2011–2020; (2) investigate the risk factors associated with local progression in the form of time to local progression (TTLP) and local progression-free survival (LPFS); (3) determine whether RT-related parameters were correlated with the occurrence of local progression in patients treated with non-palliative intent; (4) calculate the overall survival (OS) for patients based on age, performance status, and intent of RT.

### 2.5. Statistical Analysis

The Wilcoxon rank-sum and Pearson’s chi-square tests were used to compare continuous and categorical factors between patients who experienced local progression and those who did not, respectively.

Median follow-up was estimated by Kaplan–Meier analysis using overall survival data with the reversed meaning of the status indicator, i.e., we used the time from date of diagnosis to the last follow-up or death (censored). TTLP was calculated from the day of the first RT to the last follow-up (censored), death without local progression of the irradiated tumor (censored), or confirmed local progression. LPFS was calculated from the day of the first RT to the last follow-up (censored), death without local progression of the irradiated tumor, or confirmed local progression. OS was calculated from the date of osteosarcoma diagnosis to the last follow-up (censored) or death.

The Kaplan–Meier method was used to estimate survival. Multivariate and univariate Cox proportional hazard models were used to estimate hazard ratios (HR). All *p*-values < 0.05 were considered significant. Statistical analyses were performed using R version 3.6.3 software (R Foundation for Statistical Computing, Vienna, Austria).

## 3. Results

### 3.1. Patients Characteristics

A total of 126 patients with osteosarcoma who received 181 RT treatments were identified. Among them, 34 were younger than 18 years of age. The patients’ and treatments’ characteristics are presented in Table 1. The median follow-up duration was 108.9 months, with minimum and maximum duration of 1.9 and 226.8 months, respectively.

### 3.2. Radiotherapy Characteristics

The vast majority of RT treatments were performed with palliative intent (*n* = 111, 61.3%), mostly applied for locally recurrent or metastatic lesions (83.8%). The other indications for RT included the definitive treatment of unresectable disease (*n* = 13, 7.2%), neoadjuvant treatment (*n* = 3, 1.7%), adjuvant treatment after microscopically radical resection (*n* = 4, 2.2%), adjuvant treatment after non-radical resection (*n* = 21, 11.6%), oligometastatic disease (*n* = 27, 14.9%), and oligoprogressive disease (*n* = 2, 1.1%). In total, 55.8% of patients were in a good performance status at the moment of RT (ECOG 0-1). The majority of RT treatments were applied to metastatic lesions (*n* = 106, 58.6%). The other patients were irradiated due to primary (*n* = 44, 24.3%) or recurrent tumors (*n* = 31, 17.1%). The most frequently irradiated sites were pelvis (*n* = 42, 23.2%), thorax (*n* = 41, 22.7%), and spine (*n* = 38, 21%). Forty-one RT treatments (22.7%) were given concomitantly with a systemic treatment. RT was planned and delivered using a simple two-dimensional technique (*n* = 68, 37.6%), a three-dimensional conformal technique (*n* = 53, 29.3%), intensity-modulated radiotherapy (IMRT)/volumetric modulated arc therapy (VMAT) (*n* = 39, 21.5%), stereotactic techniques (*n* = 18, 9.9%), or protons (*n* = 3, 1.7%). The fraction doses ranged between 1.1 Gy and 20 Gy. The total doses ranged between 11 Gy and 70 Gy. The median EQD2 in the whole group was equal to 26.7, within the range of 9.3–240 Gy. In four cases, RT was temporarily interrupted due to RT-related toxicity.

We found a significant difference in the utilized RT techniques between RT treatments performed in the years 2000–2010 and those performed in the years 2011–2020, regardless of treatment intent. Moreover, we observed a significant difference in median EQD2 for patients treated with palliative intent between the two consecutive decades. We did not find differences in the other analyzed parameters (Table 2). RT was well tolerated. Early toxicity included mild (grade 1 and 2) and moderate (grade 3) skin and mucosal reactions. Late toxicity was mostly associated with fibrosis. We did not observe the development of secondary cancers after RT for osteosarcomas in long-term survivors. The summary of the identified toxicities is presented in Table S1.

### 3.3. Response to Radiotherapy

Complete response, partial response, and stable disease after RT, according to RECIST 1.1 or clinical assessment, were observed in 19 (10.5%), 13 (7.2%), and 131 (72.4%) cases, respectively. Progressive disease as the best response after RT occurred in 18 (9.9%) cases. In-field local progression at any time, regardless of the primary response to RT, was observed after 37 (20.4%) RT treatments.

**Figure 1:**
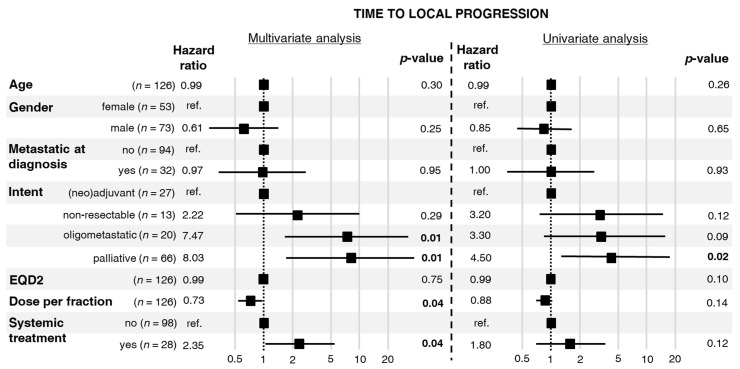
Hazard ratios for local progression with 95% confidence intervals and *p*-values calculated from a multivariate and a univariate Cox proportional hazards model.

Median TTLP was 5.3 years, range 1–221 months, see Figure S1. In the univariate analysis of factors related to the first RT, we found an influence of the palliative intent of RT on the HR of in-field local progression (Figure 1). In the multivariate analysis, intent of RT and concomitant systemic treatment were associated with a higher risk of in-field local progression, whereas a higher fraction dose reduced the risk of in-field local progression (Figure 1). The proportionality assumption required for Cox analysis was fulfilled for all the studied factors.

**Figure 2:**
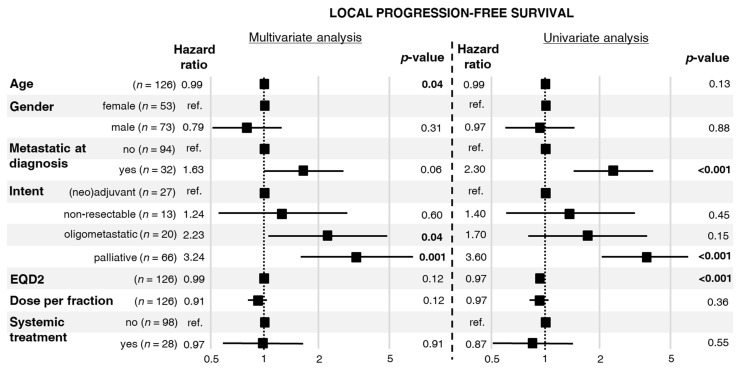
Hazard ratios for local progression or death with 95% confidence intervals and *p*-values calculated from a multivariate and univariate Cox proportional hazards model.

Median LPFS was 10 months, range 1–221 months, see Figure S2. In the univariate analysis of factors related to the first RT, distant metastases at diagnosis and palliative intent of RT increased the risk of local progression or death (Figure 2). In turn, a higher EQD2 was associated with lower risk of local progression or death. In the multivariate analysis, RT applied for oligometastatic/oligoprogressive disease and RT with palliative intent increased the risk of local progression or death.

The proportionality assumption required for Cox analysis was fulfilled for all the studied factors.

In a subgroup of patients who received RT with non-palliative intent (*n* = 70, 38.7%), in-field local progression was diagnosed in 14 (20%) cases. In this subgroup, we found significant difference in the indications for RT between patients who did and those who did not experience in-field local progression (Table 3).

### 3.4. Survival

**Figure 3:**
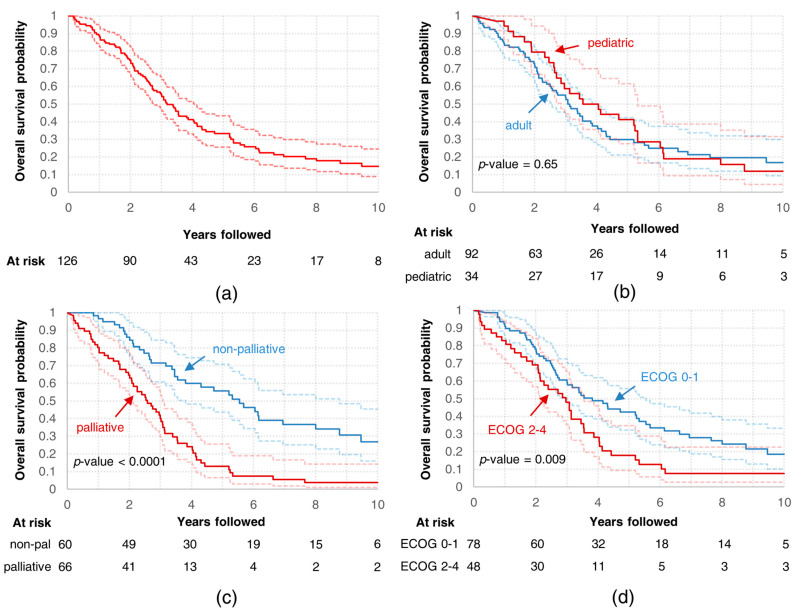
Overall survival curves for (**a**) all analyzed patients; all analyzed patients divided into subgroups based on (**b**) age, (**c**) intent of radiotherapy, and (**d**) performance status.

At the moment of the analysis, 29 patients were alive (23%). Median OS was 3.2 years (Figure 3a). Five-year OS reached 33% (95% confidence interval (CI): 26%–43%). There was no difference in median OS between pediatric and non-pediatric patients (*p* = 0.65; Figure 3b). Median OS differed between patients who underwent treatment with non-palliative intent and those treated with palliative intent (5.5 vs. 2.6 years, *p* < 0.0001; see Figure 3c), and between patients in better performance status (ECOG 0–1) and patients in performance status corresponding to ECOG 2–4 (3.8 vs. 3 years, *p* = 0.0088; see Figure 3d).

## 4. Discussion

In our study, contemporary RT techniques allowed 79.6% of local control after the first RT at the last follow-up in patients treated with both palliative and non-palliative intent. These results seem to be better than data regarding local control presented in similar analysis performed on 100 patients irradiated for osteosarcoma between 1980 and 2007 [10]. The authors reported local control for the whole group equal to 30% with 59/100 cases of in-field local progression, whereas in our cohort, in-field local progression occurred after 37/181 RT treatments. However, in a more contemporary analysis of 23 patients who underwent palliative RT for osteosarcoma, the obtained results were akin to ours [15]. This discrepancy may be explained by the evolution of RT and surgical techniques, the availability of novel systemic treatment options, and the introduction of new therapeutic concepts such as oligometastatic and oligoprogressive disease into clinical practice. Our analysis showed that RT for osteosarcoma has evolved from simple two-dimensional and three-dimensional RT into modern highly conformal RT techniques or particle therapy. However, this has not translated into higher median EQD2 in RTs applied with non-palliative intent, which was surprisingly low (59.8 Gy). According to the recommendations of the National Comprehensive Cancer Network, the Scandinavian Sarcoma Group, and the Grupo Español de Investigación en Sarcomas, the recommended dose range in conventionally fractionated RT for the adjuvant or definitive treatment of osteosarcomas is between 56 Gy and 76 Gy [2,16,17]. The summary of the recommended doses in various clinical situations is presented in Table S2.

In turn, there is no strong evidence of s direct dose–response effect in osteosarcoma. Due to the heterogeneity of our cohort, we were not able to directly investigate the dose–response relationship for local control after RT. Nevertheless, results of univariate and multivariate analyses indirectly suggested such a dependence. The palliative intent of RT, associated with low EQD2 (Table 2), was the risk factor of in-field local progression in the univariate analysis of TTLP (Figure 1). This factor was also significant in the multivariate analysis of TTLP. Similar findings were revealed in univariate and multivariate analyses of LPFS (Figure 2). Interestingly, the aforementioned analyses showed that a higher fraction dose and EQD2 are associated with a lower local risk of in-field local progression or death. Importantly, the non-palliative intents of RT differed between patients who experienced in-field local progression and those who did not. Patients with in-field local progression more frequently received RT for high-volume or treatment-resistant disease, namely, unresectable tumors or oligoprogression. A similar dependence was observed in a study published by DeLaney et al. in which better results regarding local control were related to the extent of resection, the status of the microscopical margins, and the presence of residual disease [9].

The mechanism of osteosarcoma radioresistance and unpredictable behavior after RT is unknown. Various strategies may be developed to increase the efficacy of RT in osteosarcoma treatment. First, patients with tumors presenting an extraordinary capability to repair DNA damage and a low alpha/beta ratio should benefit from higher doses per fraction. In a study performed by Gaitan-Yanguas, the author observed that osteosarcoma cells are eradicated after the administration of more than 80 Gy in conventional fractions [18]. Interestingly, it was found that a similar effect can be achieved using a lower total dose if it is delivered in a shorter period of time. In a retrospective analysis of 44 patients treated for locally advanced osteosarcomas, patients received 35 Gy in 10 fractions followed by limb-sparing surgery [19]. The authors reported 90% of tumor necrosis in 87% of the patients after treatment, 97.5% of five-year local controls, and 48.4% of five-year OS. Perioperative hypofractionation is now extensively investigated in soft tissue sarcomas, also considered to be radioresistant tumors [20]. A particular type of hypofractionation is stereotactic body RT (SBRT) that may be also used in osteosarcoma patients in the case of oligometastases, oligoprogression, or limited-volume recurrent tumors. In our cohort, SBRT was used in 18 cases, mostly for oligometastatic disease. In a case series described by Brown et al., the authors reported an institutional experience using SBRT for advanced osteosarcoma and Ewing sarcoma. The group included nine patients with osteosarcoma who underwent SBRT for 19 lesions [6]. Surprisingly, among patients with osteosarcoma, two experienced in-field local progression in two sites treated with a relatively high EQD2. The authors concluded that SBRT is a promising modality of radiation delivery for patients with advanced osteosarcoma. However, prospective evidence is awaited. In a recently developed phase III international randomized clinical trial (Stereotactic Body Radiotherapy in Patients with Rare Oligometastatic Cancers, OligoRARE, NCT04498767), SBRT to all metastatic lesions will be offered to patients with rare oligometastatic cancers as an addition to standard of care [21]. Full results are expected within 10 years.

Another way to enhance the efficacy of RT for osteosarcoma is the use of high linear energy transfer carbon-ion RT (CIRT) that causes more double-strand breaks in DNA than photon or proton therapy, with great dose conformity [22,23,24]. It has been shown as a promising method of overcoming radioresistance of osteosarcoma cells [25]. Researchers from the Gunma University Heavy Ion Medical Center (Japan) presented a series of patients treated with CIRT for unresectable bone and soft tissue tumors, which included seven patients with osteosarcoma [7]. Two-year local control rate and OS for those patients were 100% and 46%, respectively. In another retrospective analysis, the authors analyzed a cohort of 78 patients with unresectable osteosarcomas of the trunk who received CIRT as a definitive treatment. The five-year OS and local control were 33% and 62%, respectively [26]. Better results were obtained in the case of smaller tumors (<500 cm^3^), namely, five-year OS 46% and five-year local control 88%. Similar results were obtained in an analysis of 26 pediatric patients with unresectable osteosarcomas of the trunk [8]. Three- and five-year OS were 50% and 41.7%, respectively, while three- and five-year local control were 69.9% and 62.9%, respectively. A lager tumor volume was the only parameter associated with worse OS and local control.

An interesting but rare application of RT in osteosarcoma treatment could be limb-sparing surgery using the reimplantation of extracorporeally irradiated tumor-bearing bone segments. Case reports described by Böhm et al. and Mokánszki et al. showed excellent local control and good feasibility control of such treatment [27,28]. However, it is not widely used in clinical practice.

Finally, the addition of systemic agents may enhance the efficacy of RT. Machak et al. showed that RT in combination with chemotherapy may provide better than expected local and systemic control [29]. In another study, neoadjuvant radiochemotherapy provided a higher necrosis rate than neoadjuvant chemotherapy alone, which may allow the resection of primary unresectable tumors [30]. Nevertheless, the addition of neoadjuvant RT did not improve disease-free and overall survival. Moreover, several molecular pathways related to the radioresistance of osteosarcoma cells are potential therapeutic targets. CR6-interacting factor-1 (CRIF1) promotes CDK2 nuclear translocation and leads to DNA damage repair. CIRF1 was investigated in in vitro and xenograft models [31]. It was shown that CRIF1 inhibition increases the radiosensitivity of osteosarcoma cells. Furthermore, the authors of another research hypothesized that a radioresistant subpopulation of osteosarcoma cells has the capacity to regenerate by the NF-κB signaling pathway [32]. NF-κB promotes tumor growth after cellular stress such as radiation-induced DNA damage. Results of the performed experiment with osteosarcoma cell lines showed that parthenolide, an NF-κB signaling inhibitor, acted synergistically with RT, providing a significant reduction of the number of viable cancer cells in the analyzed samples. Other radiosensitizers are under investigation [33].

However, there is no evidence for a survival benefit from RT in osteosarcoma. Our studied group was very heterogeneous, and any results concerning the influence of RT on survival may have been heavily biased and misleading. Therefore, we calculated OS from the date of diagnosis; thus, this value cannot be compared with those reported by other studies presenting OS after RT or chemotherapy. Nonetheless, five-year OS in our study suggests that patients who received any form of RT during the treatment for osteosarcoma had poorer outcomes than the general population of patients of all ages and stages with osteosarcoma, corresponding to 33% and 60%, respectively [34]. Importantly, we showed a clear difference between TTLP (63.6 months) and LPFS (10 months), suggesting that the main cause of death of patients with osteosarcoma who receive RT is not local progression. Thus, RT may be a valuable method for selected patients with locally advanced or metastatic osteosarcoma, but it should be used as a part of a multimodality therapy.

Interestingly, we found only five patients with radiation-induced osteosarcomas who underwent secondary RT and no patients with Paget disease who developed osteosarcomas. The reason for a lower-than-expected incidence may be the fear of serious side effects caused by reirradiation; thus, radiation oncologists may rarely consider administering risky secondary RT with doubtful efficacy in osteosarcomas [27,35]. Due to the long developmental period of radiation-induced sarcomas and the limited data from previous decades, it was not possible to analyze primary RT in detail.

The study limitations include its retrospective nature that might introduce selection bias. This also brings a significant risk of incomplete or misinterpreted data. To reduce that risk, all records were reviewed by two of the co-authors. Another weakness of the study may be the clinical assessment of local response in the case of imaging data unavailability. Moreover, the heterogeneity of the analyzed group may make our cohort data of limited utility in guideline usage. Nevertheless, this study provides important data, which were lacking until now, due to the rarity of osteosarcomas and the limited use of RT for this radioresistant tumor.

## 5. Conclusions

We showed that RT of osteosarcoma has evolved from simple two-dimensional palliative irradiation into sophisticated highly conformal RT. We found that higher EQD2 and higher dose per fraction may be associated with better local control; however, this dependency was not clear. Nevertheless, the introduction of new RT techniques has not increased the delivered doses. Intent of RT, distant metastases, and concomitant systemic treatment affected the risk of local progression. After RT with non-palliative intent related to higher tumor volume, we observed more in-field local progressions. Our analysis suggests that RT may be a valuable treatment modality for selected patients with osteosarcoma. Due to the growing role of RT in the treatment of oligometastatic and oligoprogressive disease and the availability of novel systemic treatment options, further optimization of RT for osteosarcoma is warranted.

## Figures and Tables

**Table 1:** Patients’ and treatments’ characteristics.

Characteristic		Value
Age at diagnosis	Median	28.1
	Interquartile range	7.5–48.8
		Number of patients (%)
Sex	Female	53 (42.1)
	Male	73 (57.9)
Primary site	Head and neck	16 (12.7)
	Thorax	5 (4)
	Pelvis	22 (17.5)
	Upper arm	18 (14.3)
	Thigh	38 (30.2)
	Calf	15 (11.9)
	Spine	12 (9.5)
Osteosarcoma pathological subtype ^1^	Osteoblastic	38 (26.4)
	Chondroblastic	22 (15.3)
	Fibroblastic	8 (5.6)
	Intramedullary/intraosseus	3 (2.1)
	Small cell	3 (2.1)
	Extraskeletal	4 (2.8)
	High-grade surface	2 (1.4)
	Telangiectatic	3 (2.1)
	Parosteal	2 (1.4)
	Periosteal	1 (0.7)
	Postradiation	5 (3.5)
	No data	53 (36.8)
Underwent curative surgery	Yes	97 (77)
	No	29 (23)
Underwent perioperative chemotherapy	Yes	87 (69)
	No	10 (7.9)
	Not applicable	29 (23)
Distant metastases at the moment of diagnosis	Yes	32 (25.4)
	No	94 (74.6)
Number of radiotherapy treatments	1	88 (69.8)
	2	25 (19.8)
	3	11 (8.7)
	4	1 (0.8)
	6	1 (0.8)

^1^ More than one in an analyzed sample can be diagnosed.

**Table 2:** Radiotherapy parameters for patients treated with palliative and non-palliative intent.

Parameter		Palliative			Non-Palliative		
		2000–2010 *n* = 33	2011–2020 *n* = 78	*p*-Value	2000–2010 *n* = 16	2011–2020 *n* = 54	*p*-Value
Technique (%)	2D	26 (78.8)	38 (48.7)	0.006	2 (12.5)	2 (3.7)	0.044
	3D	7 (21.2)	28 (35.9)		8 (50.0)	10 (18.5)	
	IMRT/VMAT	0	12 (15.4)		3 (18.8)	24 (44.4)	
	SBRT/SRS	0	0		3 (18.8)	15 (27.8)	
	PT	0	0		0	3 (5.6)	
Target volume (%)	Primary tumor	2 (6.1)	16 (20.5)	0.093	10 (62.5)	16 (29.6)	0.055
	Recurrent tumor	5 (15.2)	16 (20.5)		1 (6.2)	9 (16.7)	
	Metastatic lesion(s)	26 (78.8)	46 (59.0)		5 (31.2)	29 (53.7)	
Site (%)	CNS	0	10 (12.8)	0.084	1 (6.2)	2 (3.7)	0.820
	Head and neck	2 (6.1)	2 (2.6)		3 (18.8)	12 (22.2)	
	Thorax	10 (30.3)	10 (12.8)		4 (25.0)	17 (31.5)	
	Abdomen	0	0		0	2 (3.7)	
	Pelvis	9 (27.3)	19 (24.4)		5 (31.2)	9 (16.7)	
	Spine	7 (21.2)	20 (25.6)		3 (18.8)	8 (14.8)	
	Upper extremity	4 (12.1)	8 (10.3)		0	1 (1.9)	
	Lower extremity	1 (3.0)	9 (11.5)		0	3 (5.6)	
Median EQD2 in Gy (IQR)		26.7 (6–26.7)	26.7 (6.7–26.7)	0.044 *	42 (41.1–50.6)	59.8 (38.6–70)	0.071
Median fraction dose in Gy (IQR)		4 (4–8)	4 (4–4)	0.302	3 (2.8–4)	3 (2–5)	0.815
Concurrent systemic treatment (%)	Yes	6 (18.2)	19 (24.4)	0.643	1 (6.2)	15 (27.8)	0.144
	No	27 (81.8)	59 (75.6)		15 (93.8)	39 (72.2)	

* Statistical difference driven by the significant difference in the distribution shape. Abbreviations: 2D—two-dimensional radiotherapy; 3D—three-dimensional conformal radiotherapy; CNS—central nervous system; EQD2—equivalent dose in 2 Gy fractions; IMRT—intensity-modulated radiotherapy; IQR—interquartile range; PT—proton therapy; SBRT—stereotactic body radiotherapy; SRS—stereotactic radiosurgery; VMAT—volumetric modulated arc therapy.

**Table 3:** Occurrence of in-field local progression in patients who underwent non-palliative radiotherapy.

Parameter		In-Field Progression *n* = 14	Without In-Field Progression *n* = 56	*p*-Value
Technique (%)	2D	0 (0.0)	4 (7.1)	0.418
	3D	2 (14.3)	16 (28.6)	
	IMRT/VMAT	8 (57.1)	19 (33.9)	
	SBRT/SRS	3 (21.4)	15 (26.8)	
	PT	1 (7.1)	2 (3.6)	
Intent (%)	Adjuvant R0	0 (0.0)	4 (7.1)	0.02
	Adjuvant R1/R2	3 (21.4)	18 (32.1)	
	Definitive unresectable	5 (35.7)	8 (14.3)	
	Neoadjuvant	0 (0.0)	3 (5.4)	
	Oligometastatic	4 (28.6)	23 (41.1)	
	Oligoprogressive	2 (14.3)	0 (0.0)	
Site (%)	CNS	0	3 (5.4)	0.734
	Head and neck	4 (28.6)	11 (19.6)	
	Thorax	3 (21.4)	18 (32.1)	
	Abdomen	1 (7.1)	1 (1.8)	
	Pelvis	4 (28.6)	10 (17.9)	
	Spine	2 (14.3)	9 (16.1)	
	Upper extremity	0	1 (1.8)	
	Lower extremity	0	3 (5.4)	
Median EQD2 in Gy (IQR)		60 (4–70)	50 (38.3–68.4)	0.566
Median fraction dose in Gy (IQR)		2.5 (2–4.8)	3 (2.2–5)	0.153
Concurrent systemic treatment (%)	Yes	5 (35.7)	11 (19.6)	0.355
	No	9 (64.3)	45 (80.4)	

## Data Availability

All data generated or analyzed during this study are available upon reasonable request.

## Supplementary Materials

The following are available online at https://www.mdpi.com/2073-4409/10/2/366/s1, Table S1: The summary of identified radiotherapy toxicities in the analyzed cohort of patients with osteosarcoma, Table S2: Recommended total doses in 2 Gy fractions for definitive treatment in patients with localized osteosarcoma, Figure S1: Time to local progression, Figure S2: Local progression-free survival.
