# Supplementary figures and images for: Radiotherapy in the Management of Pediatric and Adult Osteosarcomas: A Multi-Institutional Cohort Analysis
Source: Cells. 2021 Feb 10;10(2):366. doi: 10.3390/cells10020366 (PMC7916348; doi:10.3390/cells10020366)

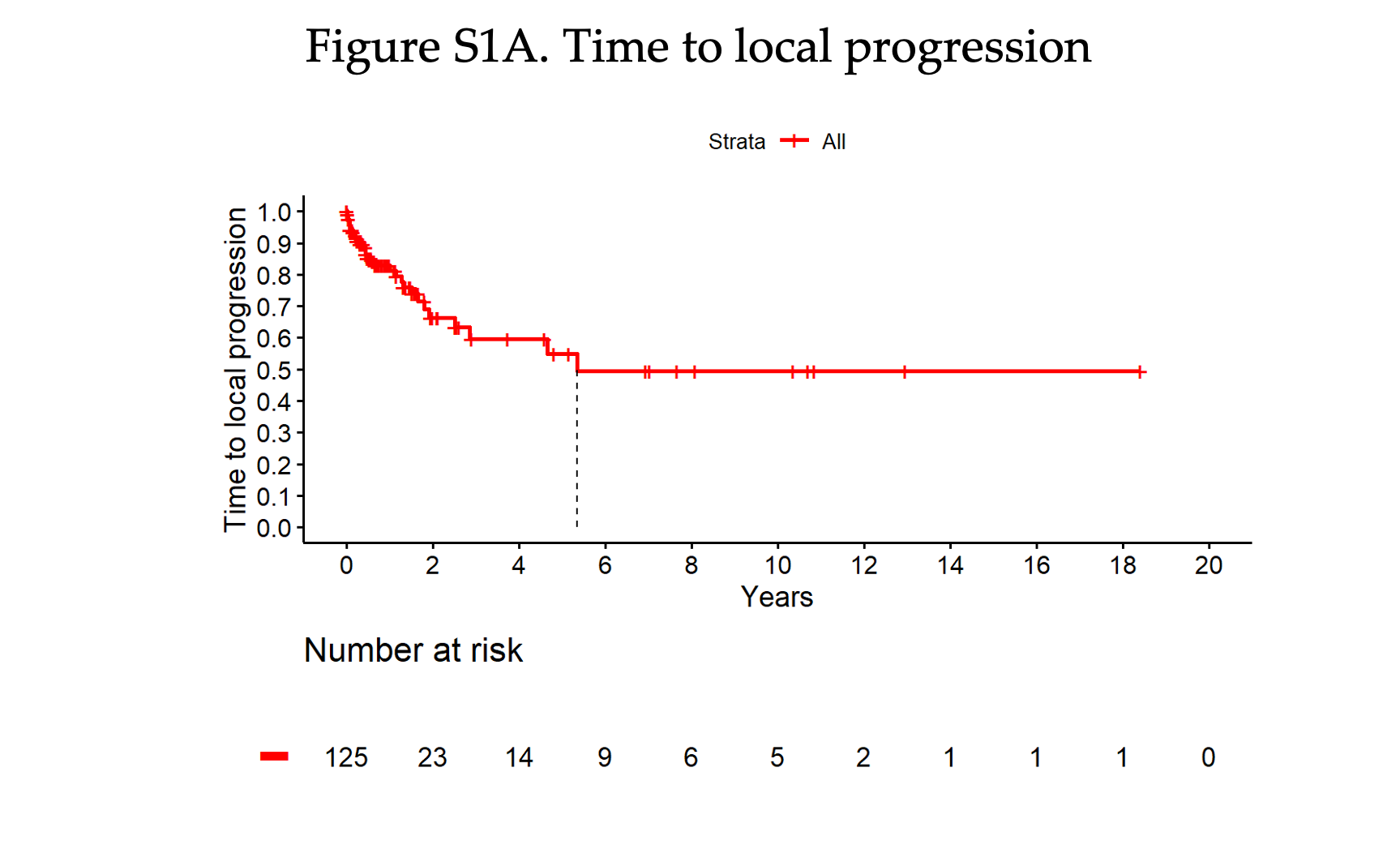

Supplement: Supplementary file 1 [file cells-10-00366-s001.zip › Figure_S1A.png]

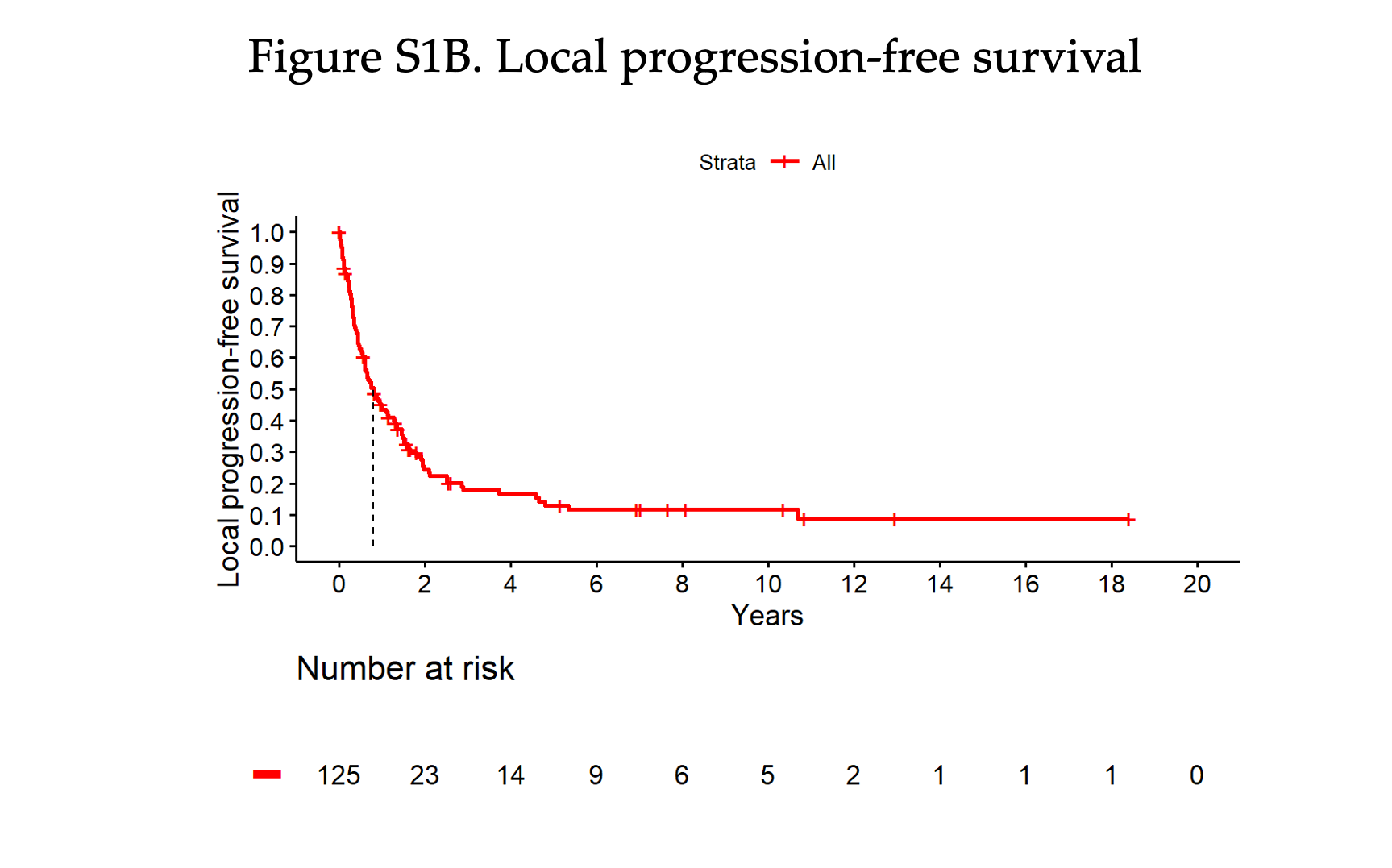

Supplement: Supplementary file 1 [file cells-10-00366-s001.zip › Figure_S1B.png]
